# Orthogonal Design Study on Factors Affecting the Determination of Common Odors in Water Samples by Headspace Solid-Phase Microextraction Coupled to GC/MS

**DOI:** 10.1155/2013/340658

**Published:** 2013-08-13

**Authors:** Shifu Peng, Zhen Ding, Weiwen Xia, Hao Zheng, Yuting Xia, Xiaodong Chen

**Affiliations:** ^1^School of Public Health, Southeast University, Nanjing, Jiangsu 210009, China; ^2^Department of Environmental and Endemic Diseases Control, Jiangsu Provincial Center for Disease Control and Prevention, Nanjing, Jiangsu 210009, China; ^3^Department of Physical and Chemical Test, Jintan City Center for Disease Control and Prevention, Changzhou, Jiangsu 213200, China

## Abstract

Geosmin and 2-MIB are responsible for the majority of earthy and musty events related to the drinking water. These two odorants have extremely low odor threshold concentrations at ng L^−1^ level in the water, so a simple and sensitive method for the analysis of such trace levels was developed by headspace solid-phase microextraction coupled to gas chromatography/mass spectrometry. In this study, the orthogonal experiment design L_32_ (4^9^) was applied to arrange and optimize experimental conditions. The optimum was the following: temperatures of extraction and desorption, 65°C and 260°C, respectively; times of extraction and desorption, 30 min and 5 min, respectively; ionic strength, 25% (w/v); rotate-speed, 600 rpm; solution pH, 5.0. Under the optimized conditions, limits of detection (S/N = 3) were 0.04 and 0.13 ng L^−1^ for geosmin and 2-MIB, respectively. Calculated calibration curves gave high levels of linearity with a correlation coefficient value of 0.9999 for them. Finally, the proposed method was applied to water samples, which were previously analyzed and confirmed to be free of target analytes. Besides, the proposal method was applied to test environmental water samples. The RSDs were 2.75%~3.80% and 4.35%~7.6% for geosmin and 2-MIB, respectively, and the recoveries were 91%~107% and 91%~104% for geosmin and 2-MIB, respectively.

## 1. Introduction

Musty and earthy odors are troublesome in water samples (such as tap water, reservoirs, and lakes) because they dramatically influence the esthetic quality and consumer acceptability of the drinking water [1, 2]. Metabolites are produced in the process of degradation of green-blue algae, which are responsible for this malodor in water, especially during the period of algae blossom in summer [3–5]. The chemical by-products, geosmin (*trans-1,10-dimethyl-trans-9-decalol*) and 2-MIB (*2-methylisoborneol*), are commonly found in lakes and reservoirs, where people can smell the odor of these compounds in the drinking water even at the determination of 10 ng L^−1^ or less, but it would be difficult to identify and quantify these two trace volatile organic compounds (VOCs) [6–10]. Therefore, how to extract and enrich them tends to be the key step for qualitative and quantitative analysis at this trace level.

To date, a variety of techniques for extraction and enrichment have been established and applied for analyzing earthy and musty compounds. Among these techniques, closed-loop stripping analysis (CLSA) and some of its modified versions have been widely used for the extraction of trace amounts of malodor such as geosmin and 2-MIB in water samples. The result showed that CLSA was a good tool for the analysis of geosmin and 2-MIB at a low-level threshold [11]. However, it is labor-intensive and time-consuming. Some other methods, such as purge and trap coupled to gas chromatography with mass spectrometry [12, 13] or to GC-FID [14], liquid-liquid microextraction (LLME) [15], stir bar sorptive extraction (SBSE) [16–18], and solid-phase extraction (SPE) [19], can also be taken to detect the VOCs in water at  ng L^−1^ levels. Although these techniques greatly improve the limits and sensitivity of detection, some shortcomings such as being unsuitable for the analysis of low-boiling-point odors and time-consuming (SPE, SBSE) [20, 21] and lacking the stability of droplet during extraction (LPME) restrict the extensive use of these methods. As technology advances, solid-phase microextraction (SPME) was first developed, and it was reported that headspace SPME (HS-SPME) was effective for collecting volatile organic compounds from Penicillium [22]. HS-SPME has become the most popular technique in pretreating and enriching a variety of water odors [23–27], because of requiring no solvents during extraction by HS-SPME, which cannot be achieved by LLME; being a simpler operation if compared with other methods as SPE, CLSA, and SBSE; and, the most important merit, being able to enrich the target selectively by suitable fiber, which cannot be obtained by SPE and LLME.

It had been reported that the efficiency of HS-SPME was subject to several factors such as extraction temperature, ionic strength, and rotate-speed. The method of “one factor at a time”, the traditional method for optimizing experiment conditions of HS-SPME or purge and trap (P&T), was to change the level of one factor while keeping others constant [12, 24, 26, 28], which was almost the only one to optimize these factors in current reports for determining trace VOCs in water samples. Obviously, this method is time-consuming when these factors and their levels reach a certain number. In addition, it often overlooks the interactions among the factors, despite having no interactions in this study. In this paper, however, the orthogonal experiment design was applied to arrange and optimize experiment conditions, including extraction temperature, desorption temperature, extraction time, solution pH, ionic strength, and rotate-speed. Besides, the process of mass spectrometry was optimized to improve the sensitivity and efficiency during the detection and analysis in this study. The good figures of merit for the analysis of the trace amount of odors in water samples have been obtained by combining them. Finally, the analytical method has been validated and applied to test on water samples.

## 2. Experimental

### 2.1. Chemicals and SPME Apparatus

Two categories of compounds commonly observed in water, geosmin and 2-MIB, and the internal standard *2-isobutyl-3-methoxypyrazine* (IBMP) were obtained from Sigma-Aldrich (USA) at a concentration of 100 mg L^−1^ in methanol, 1 mg L^−1^ mixed standard solutions of the two target compounds in methanol, and all of them were stored in the dark at 4°C. The details of the three compounds are shown in Table 1.

Deionized water was prepared on a water purification system (Gradient A10) supplied by Millipore (Billerica, MA, USA). Sodium chloride (analytical grade, China), which was added to the samples before extraction, was conditioned by heating at 450°C for 4 h before use. The SPME apparatus was purchased from Supelco (USA), including fiber 30/50 *μ*m DVB/CAR/PDMS (no. 57348-U) [26, 29], fiber holder, 60 mL specialized vials for SPME, sampling stand, magnetic stirrer, and injection catheter (no. 57356-U).

### 2.2. SPME Procedures

The extraction conditions shown in Table 3 were followed. After putting NaCl and a stir bar in a 60 mL vial, 40 mL of mixed standard solutions (50 ng L^−1^) for orthogonal experiment, or 40 mL of environmental water samples, was added, and 2 *μ*L IBMP (1 mg L^−1^) was added to every sample. The vial was sealed with polytetrafluoroethylene (PTFE) septum cap and placed in a water bath. Several minutes after the temperature was achieved in the vial, the outer needle of fiber was used to penetrate the septum, and the fiber was exposed to the headspace for extraction. After exposure, the fiber was immediately inserted into the GC injection port for desorption.

### 2.3. Gas Chromatography-Mass Spectrometry

A Varian 300 GC/MS/MS (Varian, Inc., CA, USA) with ion trap and mass spectrometer was obtained with a Varian VF-5 MS capillarity column (30 m × 0.25 mm × 0.5 *μ*m). The temperature of the injector was 260°C adjusted to splitless mode. The carrier gas was helium at a flow of 1 mL min^−1^. The temperature of the oven started at 40°C and was held for 5 min. Then, the temperature was 8°C min^−1^ to achieve 160°C (total time: 20 min) followed by 20°C min^−1^ to achieve 260°C (total time: 25 min). The electron impact (EI) MS conditions were as follows: ion-source temperature: 230°C; MS transfer line temperature: 250°C; solvent delay time: 5 min; and ionizing voltage: 70 eV. The mass spectrogram in full-scan mode was obtained at the *m*/*z* range of 80–200 u. According to the MS scan function (SIM mode), the process was divided into three main segments as shown in Table 2. The method of internal standard was applied to construct the calibration curve and determine the concentrations of 2-MIB and GSM in water.

## 3. Results and Discussion

**Figure 1:**
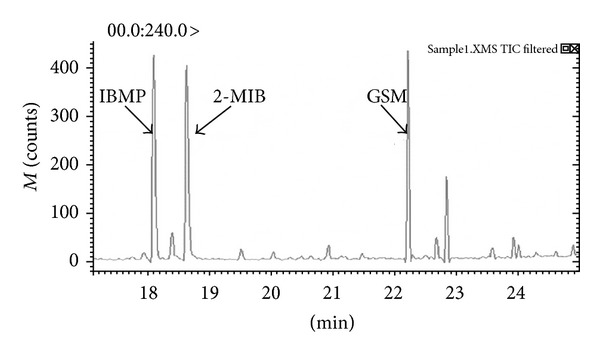
To identify the two odors and the internal standard compound by HS-SPME.

To obtain the qualitative and quantitative ions, the two target analytes and the internal standard compound were first identified simultaneously by HS-SPME/GC-MS in the scan mode. The selected ions and retention time (*t*_*R*_) are listed in Table 2. The chromatogram (full-scan mode) is shown in Figure 1, and the full-scan mass spectra from 17.0 to 24.3 min were obtained with *m*/*z* range of 80–200 u.

### 3.1. Optimization of Headspace Solid-Phase Microextraction and Desorption

In order to optimize the extraction of GSM and 2-MIB by HS-SPME, several parameters were investigated. The orthogonal design experiment is a valid approach by applying the orthogonal table L_*n*_ (*m* ^*k*^) to arrange experiments. In the orthogonal table, *n* represents the number of experiments; *m* is the number of factor levels; and *k* is the number of factors. The multiple factors as well as the multiple levels can be dealt with by this design, and it also can obtain the optimal design with less experimental effort. And the experimental results can then be calculated by the analysis of variance, which can identify the main significant factors and levels. This method had been applied to optimize experimental conditions for determining the several phytohormones in natural coconut juice by Wu and Hu [31], and it has been used by Huang et al. [32] for the determination of glycol ethers it had been also extraction of fat-soluble vitamins by Sobhi et al. [33] and analysis of 17 organochlorine pesticides in water samples by Qiu and Cai [34]. In this study, utilized orthogonal experiment design L_32_ (4^9^) was applied to arrange and optimize experiment conditions. The concrete experiments are listed in Table 3, and, all experiments were repeated twice, and then, the average peak area was applied to weigh the efficiency of HS-SPME under different conditions.

According to the analytical results calculated by SPSS 19.0 in Table 4, for the maximum for 2-MIB was K31, K42, K43, K34, K45, K46, and K47, and that for GSM was K41, K42, K43, K34, K45, K46, and K27. In other words, the optimum conditions for 2-MIB were listed as follows: temperatures of extraction and desorption: 60°C and 260°C, respectively; times of extraction and desorption: 40 min and 7 min, respectively; ionic strength: 30% (w/v); rotate-speed: 600 rpm; and solution pH: 8.0. And those for GSM were listed as follows: temperatures of extraction and desorption: 70°C and 260°C, respectively; times of extraction and desorption: 40 min and 7 min, respectively; ionic strength: 25% (w/v); rotate-speed: 600 rpm; and solution pH: 6.0.

Table 5 shows the results of the analysis of variance (ANOVA) for the significance of the main factors, which were based on the peak area of headspace in simulative water samples. As to 2-MIB, extraction temperature, extraction time, desorption time, and ionic strength were significant factors (*P* ≤ 0.01), while other factors did not have a significant effect (*P* ≥ 0.05) within the studied range. For the GSM, only extraction temperature and extraction time were significant factors (*P* ≤ 0.01), while others were not significant (*P* ≥ 0.05).

### 3.2. Effects of Extraction Temperature, Extraction Time, Ionic Strength, and Desorption Time on Responses

**Figure 2:**
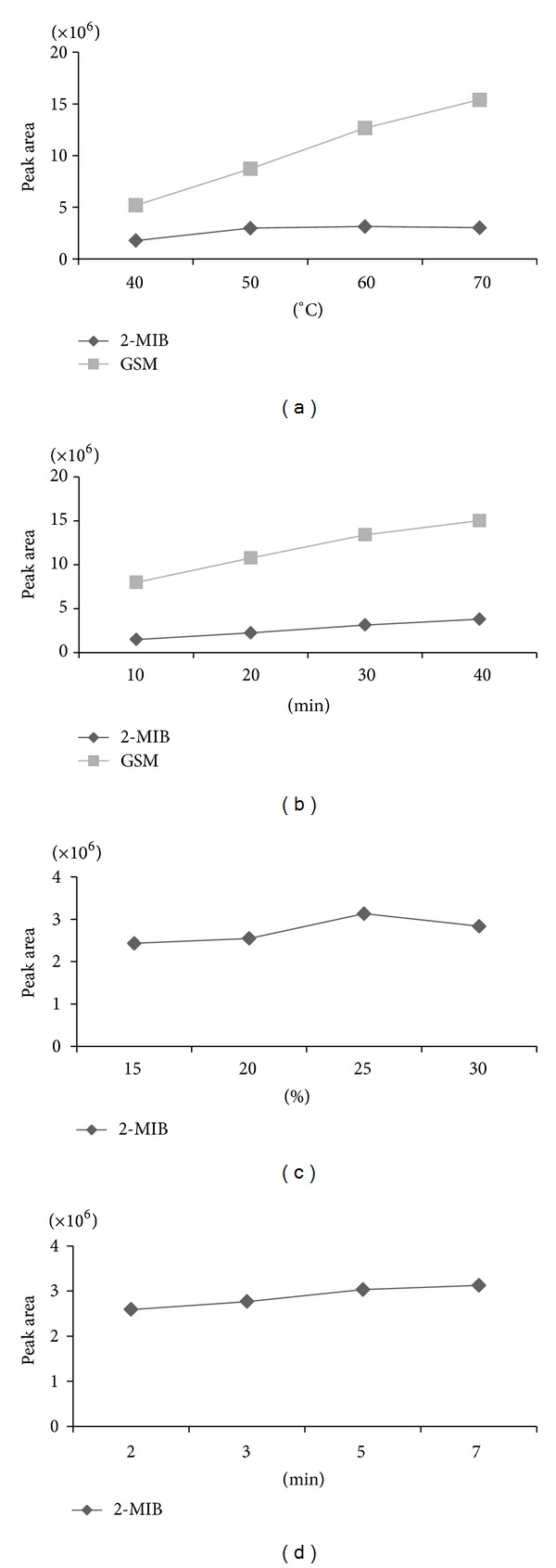
The effects of extraction temperature, extraction time, ionic strength, and desorption time on responses. (a) extraction temperature for GSM and 2-MIB; (b) extraction times for GSM and 2-MIB; (c) ionic strength for 2-MIB; and (d) desorption time for 2-MIB.

According to the results of ANOVA of the previous main factors, the significant factors were studied at 10 ng L^−1^ of mixed standard solutions by using the method of “one factor at a time.” Each of them was tested twice, and the results were obtained in Figure 2.

#### 3.2.1. Effect of Extraction Temperature

Responses were calculated upon the conditions of 40°C, 50°C, 60°C, and 70°C extraction temperatures (time of extraction and desorption: 30 min and 5 min, resp.; ionic strength: 25% (w/v); rotate-speed: 600 rpm; desorption temperature: 260°C; the solution pH: 5.0). As shown in Figure 2(a), we studied the SPME analyses run at the selected temperature, the extraction efficiency of the two analytes increased as the extraction temperature was, from 40°C to 60°C while a decrease was observed for 2-MIB and a slow increase was observed for GSM between 60°C and 70°C as reported similarly by Saito et al. [24]. The most likely reasons can be concluded as follows: firstly, as the extraction temperature was growing, the increasing amount of water vapor would be assembled on the fiber, which would lower the extraction efficiency; secondly, the different molecular weight of VOCs was known to be inconsistently susceptive to the extraction fiber [35]; thirdly, this can be explained by the partition coefficient between the fiber and the analytes. In other words, *K*_fs_ = *K*_0_exp⁡[−Δ*H*/*R*(1/*T* − 1/*T*_0_)] [36]; that is to say, the partition coefficient (*K*_fs_) would be altered if the extraction temperature was changed from *T*_0_ to *T*, because the potential energy of the analyte on the coating material will be less than its potential energy in the sample if the *K*_fs_ is more than one. Therefore, the value of *K*_fs_ will decrease as the extraction temperature is increasing, which can result in the decreased extraction efficiency as reported by Chai and Pawliszyn [37]. When considering the extraction temperature, 65°C was the optimal choice as obtained in Figure 2(a).

#### 3.2.2. Effect of Extraction Time

Responses were calculated upon the conditions of 10, 20, 30, and 40 min extraction temperature times (desorption time: 5 min; ionic strength, 25% (w/v); rotate-speed: 600 rpm; extraction and desorption temperatures: 65°C and 260°C, resp.; and solution pH: 5.0). As shown in Figure 2(b), we studied the SPME analyses run at the selected time; the extraction efficiency of the two analytes increased rapidly as extraction time was from 10 min to 30 min, while a slow increase was observed for both of them between 30 and 40 min. However, the equilibrium time for this fiber may be 40 min or more, but we desired shorter extraction time to maximize the sample. Therefore, the extraction time 30 min was selected for experiments, and also this allowed the GC-MS analysis (25 min) to be performed nearly in the approximate time as in the HS-SPME procedure.

#### 3.2.3. Effect of Ionic Strength

The suitable salt addition could improve the transfer of analytes from the aqueous phase to the gaseous phase, so this can result in a higher concentration of the odors in the headspace. Responses were calculated upon the conditions of 15%, 20%, 25%, and 30% (w/v) ionic strengths (times of extraction and desorptions: 30 min and 5 min, resp.; rotate-speeds: 600 rpm; extraction, and desorption temperatures: 65°C and 260°C, resp.; and solution pH: 5.0). As shown in Figure 2(c), it was fairly clear that 25% (w/v) was most suitable for the extraction process, and this concentration of salt was selected for the future experiments.

#### 3.2.4. Effect of Desorption Time

As shown in Figure 2(d), the desorption time (2, 3, 5, and 7 min) profile is studied. The peak area of 2-MIB remained unchanged as desorption time after 5 min. In other words, 5 min was enough for desorption. Thus, 5 min was selected as the optimal time.

#### 3.2.5. Effects of Other Factors

According to the results of the analysis of variance, some factors such as rotate-speed, desorption temperature, and solution pH did not have significant effects (*P* ≥ 0.05) within the given range. Consequently, 600 rpm, 260°C, and 5.0 were chosen, respectively, for rotate-speed, desorption temperature, and solution pH [24, 26].

### 3.3. Optimization of Mass Spectrometry

**Figure 3:**
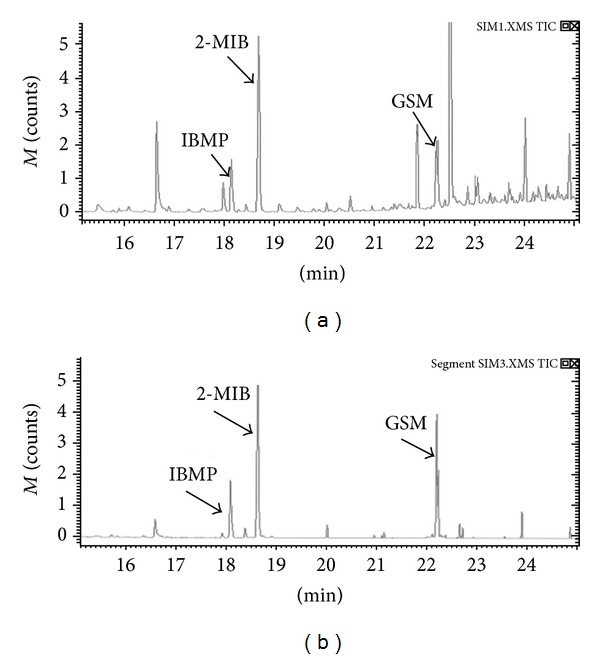
The determination of analytes in SIM mode. (a) Not using segments for the determination of analytes in SIM mode. (b) The MS scan function (SIM mode) for the determination of analytes.

The 10 ng L^−1^ spiked mixed standard solutions were detected by two methods later. As shown in Figure 3(b), the MS analysis progress in SIM mode was separated into five segments. To be more specific, segment 1 started from 15.0 min, selected ion *m*/*z* = 124, segment 2 from 18.4 min, *m*/*z* = 107, segment 3 from 19.0 min, nothing, segment 4 from 22.0 min, *m*/*z* = 112, and segment 5 from 22.4 min, nothing. In this case, the peaks can be separated effectively, and some disturbed peaks would be excluded. In contrast, as shown in Figure 3(a), it would result in some unidentified peaks by not using the segments to analyze the compounds. The signal to noise ratio (S/N) was shown in Table 6, and it was fairly clear that the MS scan function was more effective for the determination of analytes.

### 3.4. Method Validation

The proposed HS-SPME/GC-MS method had been validated in terms of accuracy, linearity, limits of detection, relative standard deviation (RSD), and recovery.

#### 3.4.1. Calibration Curves, Limits of Detection, and RSDs

Linearity was studied by extracting the two odors in mixed standard solutions at five levels, ranging from 5 to 100 ng L^−1^. Calculated calibration curves gave high levels of linearity with a correlation coefficient value of 0.9999 for both of geosmin and 2-MIB, and the RSDs were 2.75%~3.80% and 4.35%~7.6% for GSM and 2-MIB, respectively. Under the optimized experimental conditions, limits of detection (S/N = 3) and limits of quantitation (S/N = 10) for geosmin were 0.04 and 0.14 ng L^−1^, and those for 2-MIB were 0.13 and 0.42 ng L^−1^, respectively (Tables 7 and 8).

#### 3.4.2. Test on Environmental Samples

Tap water, deionized water, and lake water were used to verify the applicability of this method for the analysis of these two odors in water samples. As shown in Table 9, the high recoveries were obtained. This fairly indicated that the proposed method could be used to analyze these musty and earthy odors in water samples.

## 4. Conclusion

This study has demonstrated the application of the orthogonal experiment design for screening the significant factors of HS-SPME for the analysis of volatile organic compounds in water samples. Also, the MS scan function was an effective approach for the determination of analytes. The proposed method has been validated by excellent results (i.e., high sensitivity, low limits of detections, and high levels of linearity). Therefore, this method would be most likely to be applied to optimize factors for future study for analysis of some other odors in water (i.e., 2-isopropyl-3-methoxy pyrazine and 2,4,6-trichloroanisole).

## Figures and Tables

**Table 1:** The CAS number, molecular weight, boiling point, and odor threshold of the three compounds.

Compounds	CAS no.	Molecular weight	Boiling point^a^ (°C)	Odor threshold concentration^c^ (ng L^−1^)
GSM	19700-21-1	182	270^b^/249	4
2-MIB	2371-42-8	168	210	9
IBMP	24683-00-9	166	236	1

^a^Calculated by EPISuit v.4.10 (2011) developed by the US EPA 2011, and boiling points were obtained by the Stein and Brown method. ^b^This boiling point was obtained by EPISuit v.4.10. ^c^Detected by sensory and cited from Mallevialle and Young et al. [10, 30].

**Table 2:** The parameters of the MS scan function (SIM mode) for the determination of analytes.

Compounds	Retention time (min)	Segment (min)	Quantitative ions (*m/z*)	Secondary ions (*m/z*)
GSM	22.217	22.0–22.4	112	125
2-MIB	18.663	18.4–19.0	107	95,135
IBMP	18.121	15.0–18.4	124	94,151

**Table 3:** The experimental design based on Taguchi's L_32_ (4^9^) orthogonal array and the response of the peak area count by GC-MS^a^.

Exp. no. and order	Factors^b^							Responses^c^ (peak area ×10^7^)	
	A	B	C	D	E	F	G	2-MIB	GSM
1	40	10	300	15	2	200	5	0.462	1.305
2	40	30	500	25	2	240	5	1.782	5.285
3	40	40	600	15	3	220	6	2.214	7.223
4	40	20	400	25	3	260	6	2.155	8.261
5	40	30	500	30	5	220	7	1.843	6.323
6	40	10	300	20	5	260	7	0.734	2.423
7	40	20	400	30	7	200	8	2.013	6.054
8	40	40	600	20	7	240	8	2.234	7.568
9	50	40	500	20	3	200	5	2.971	8.734
10	50	20	300	30	3	240	5	2.225	8.334
11	50	10	400	20	2	220	6	0.835	2.568
12	50	30	600	30	2	260	6	2.644	8.467
13	50	20	300	25	7	220	7	2.325	8.407
14	50	40	500	15	7	260	7	3.173	9.462
15	50	30	600	25	5	200	8	2.828	8.532
16	50	10	400	15	5	240	8	1.122	3.586
17	60	10	600	30	7	220	5	2.587	7.256
18	60	30	400	20	7	260	5	3.105	9.637
19	60	40	300	30	5	200	6	3.235	11.38
20	60	20	500	20	5	240	6	3.013	8.467
21	60	30	400	15	3	200	7	3.089	9.435
22	60	10	600	25	3	240	7	2.793	7.315
23	60	20	500	15	2	220	8	2.958	8.316
24	60	40	300	25	2	260	8	3.273	11.49
25	70	40	400	25	5	220	5	2.843	10.78
26	70	20	600	15	5	260	5	2.772	15.85
27	70	10	500	25	7	200	6	2.336	9.886
28	70	30	300	15	7	240	6	3.131	13.41
29	70	20	600	20	2	200	7	2.563	13.27
30	70	40	400	30	2	240	7	2.656	12.78
31	70	30	300	20	3	220	8	2.895	12.92
32	70	10	500	30	3	260	8	2.324	8.737

^a^In this table, the error term was not listed. ^b^Factor A: extraction temperature (°C); Factor B: extraction time (min); Factor C: rotate-speed (rpm); Factor D: ionic strength (w/v, %); Factor E: desorption time (min); Factor F: desorption temperature (°C); and Factor G: solution pH. ^c^Peak area was calculated by quantitative ions under SIM mode, and area rejection: 10,000; initial threshold: 1; and peak width: 0.04.

**Table 4:** The basic analytical results of the orthogonal experiment design L_32_ (4^9^).

Compounds (×10^7^)	*K* value	Factors^a^						
		A	B	C	D	E	F	G
2-MIB	*K*1	13.437	13.193	18.280	18.921	17.173	19.497	18.747
	*K*2	18.123	20.024	17.818	18.350	20.666	18.500	19.563
	*K*3	24.053	21.317	20.400	20.335	18.390	18.956	19.176
	*K*4	21.520	22.599	20.635	19.527	20.904	20.180	19.647

GSM	*K*1	44.442	43.076	69.669	68.587	63.481	68.596	67.181
	*K*2	58.090	76.959	63.101	65.587	70.959	63.793	69.662
	*K*3	73.296	74.009	65.210	69.956	67.341	66.745	69.415
	*K*4	97.633	79.417	75.481	69.331	71.680	74.327	67.203

^a^Factor A: extraction temperature (°C); Factor B: extraction time (min); Factor C: rotate-speed (rpm); Factor D: ionic strength (w/v, %); Factor E: desorption time (min); Factor F: desorption temperature (°C); and Factor G: solution pH.

**Table 5:** The analysis of the variance of the main factors on the respective peak area of headspace volatile odors in simulated water samples.

Source^a^	2-MIB					GSM				
	df^b^	SUM^c^	MS^d^	*F*-value	Significant	df	SUM	MS	*F*-value	Significant
A	3	7.90979	2.63659	100.8	∗∗	3	194.85201	64.95067	56.1	∗∗
B	3	6.59627	2.19875	84.1	∗∗	3	108.42397	36.14132	31.2	∗∗
C	3	0.77847	0.25949	9.9	∗	3	11.25019	3.75006	3.2	
D	3	0.27097	0.09032	3.45		3	1.40387	0.46795	0.4	
E	3	1.22373	0.40791	15.6	∗∗	3	5.32751	1.77583	1.5	
F	3	0.19630	0.06543	2.50		3	7.39079	2.46359	2.1	
G	3	0.06370	0.02123	0.81		3	0.69210	0.23070	0.2	
Error	10	0.26148	0.02614			10	11.56231	1.15623		

∗ and ∗∗: significant at *P* ≤ 0.01 and *P* ≤ 0.001, respectively. ^a^Source A: extraction temperature (°C); Source B: extraction time (min); Source C: rotate-speed (rpm); Source D: ionic strength (w/v, %); Source E: desorption time (min); Source F: desorption temperature (°C); and Source G: solution pH. ^b^Degree of freedom; ^c^sum of square; and ^d^mean of square.

**Table 6:** The comparison of the signal to noise ratio (S/N) by two methods.

Compounds	Quantitative ion	Concentration (ng L^−1^)	S/N_1_^a^	S/N_2_^b^	(S/N_1_)/(S/N_2_)
IBMP	124	10	6899	1731	3.98
2-MIB	107	10	2046	906	2.26
GSM	112	10	737	144	6.46

^a^S/N_1_ was obtained by not using segments in SIM mode. ^b^S/N_2_ was obtained by the MS scan function in SIM mode.

**Table 7:** The calibration curves and limits of detection for 2-MIB and GSM.

Compounds	Calibration curves	Correlation coefficients (*R*)	LOD (ng L^−1^)	LOQ (ng L^−1^)
2-MIB	$\hat{y}=0.1358x+0.0334$	0.9999	0.13	0.4
GSM	$\hat{y}=1.6583x+0.3079$	0.9999	0.04	0.2

**Table 8:** The relative standard deviations (RSDs)^a^ for 2-MIB and GSM.

Exp. no.^b^	2-MIB		GSM	
	20 ng L^−1^	100 ng L^−1^	20 ng L^−1^	100 ng L^−1^
1	24.2	93.8	19.3	102.1
2	24.3	90.1	20.5	100.7
3	23.2	102.0	20.6	102.1
4	22.6	95.1	21.9	100.6
5	22.1	94.0	20.6	106.7
6	20.2	91.0	20.4	105.7
7	20.2	91.2	19.8	107.2
RSD (%)^a^	7.6	4.35	3.8	2.75

^a^Using IBMP as the internal standard. Compound concentration: 10 ng L^−1^. ^b^Spiked de-ionized water sample.

**Table 9:** The recovery of environmental samples.

Compounds	Samples	Concentration	Test results of spiked samples (ng L^−1^)		Recovery (%)	
		(ng L^−1^)	20 ng L^−1^	100 ng L^−1^	20 ng L^−1^	100 ng L^−1^
2-MIB	Deionized water	<0.2	20.8	92.0	104	92.0
	Tap water	1.8	23.9	95.3	110	93.5
	Lake water	2.9	23.6	94.0	103	91.1

GSM	Deionized water	<0.4	21.4	103.7	107	104
	Tap water	1.9	20.1	101.6	90.8	99.7
	Lake water	2.6	22.1	104.8	97.3	102
